# Prevalence and risk factors for high-risk human papillomavirus infection among women from three southern geopolitical zones of Nigeria

**DOI:** 10.3389/fonc.2023.1254304

**Published:** 2023-10-09

**Authors:** Chika Kingsley Onwuamah, Ning Feng, Abidemi Esther Momoh, Mabel Uwandu, Rahaman Ademolu Ahmed, Ifeoma Idigbe, Grace Deborah Vincent, Chinenye Angela Ogbu, Nkem Okonkwo, Judith Sokei, Bowofoluwa Sharon Abimbola, Temiloluwa Ojopagogo, Leona Chika Okoli, Mary Adesina, Priscilla Ngozi Ezemelue, Omowunmi Sowunmi, Jane Okwuzu, Olaoniye Habeebat Labo−Popoola, Joseph Ojonugwa Shaibu, Greg Aigbe Ohihoin, Emily Nzeribe, Agatha David, Olufemi Olaleye, Ighovwerha Ofotokun, Xiao−ping Dong, Oliver Chukwujekwu Ezechi

**Affiliations:** ^1^ Centre for Human Virology and Genomics, Department of Microbiology, Nigerian Institute of Medical Research, Lagos, Nigeria; ^2^ Center for Global Public Health, Chinese Centre for Disease Control and Prevention, Beijing, China; ^3^ Division of Allergy and Clinical Immunology, Division of Genetics, Department of Medicine, Brigham and Women’s Hospital, Harvard Medical School, Boston, MA, United States; ^4^ Centre for Reproduction and Population Health Studies, Department of Clinical Sciences, Nigeria Institute Medical Research, Lagos, Nigeria; ^5^ Department of Obstetrics and Gynaecology, Delta State University Teaching Hospital, Oghara, Delta, Nigeria; ^6^ Department of Pediatric Oncology (Hematology), Washington University in St. Louis, St. Louis, MO, United States; ^7^ Department of Obstetrics and Gynaecology, Federal Medical Centre, Owerri, Imo, Nigeria; ^8^ Screening Section, Optimal Cancer Care Foundation Centre, Lagos, Nigeria; ^9^ Division of Infectious Diseases, Department of Medicine, Emory University School of Medicine, Atlanta, GA, United States

**Keywords:** HPV, risk factor, women, Nigeria, cervical cancer

## Abstract

**Introduction:**

Human Papillomavirus (HPV) infection is a risk factor for cervical cancer, the fourth most common cancer among women globally. Its burden is the highest in sub-Saharan Africa, with over 90% mortality. Interventions may fail without evidence-based data on stratified prevalence and risk factors among most at-risk women across Nigeria.

**Methods:**

A cross-sectional comparative study, with participants recruited from the Nigerian Institute of Medical Research’s Clinics, NGO outreaches, a cancer screening centre and a university teaching hospital. Questionnaires were self-administered. Trained medics performed sampling at healthcare facilities, and self-sampling was used at outreaches.

**Results:**

Nine hundred eighty-five study participants were recruited. About 37% and 27% of the women knew about HPV and its vaccines, respectively, but only 6% confirmed vaccination with HPV vaccines. HPV prevalence was highest among women with unknown marital status (35.9%), single women (33.8%), widowed/divorced/separated women (30.3%), and married/cohabiting women (19.6%). HPV infection was significantly higher among women who take alcohol (odds=1.7 [95% CI: 1.2-2.4]) and women who smoke (odds=2.6 [95% CI: 1.4 - 4.6]. HPV strains detected included HPV16 (1.3%), HPV18 (1.5%), Low Risk (0.2%) and Other High-Risk groups (19.7%).

**Conclusion:**

The inverse relationship between prevalence and education suggests interventions improving awareness and prevention would be impactful. Such interventions could also target HIV-positive women, women presenting with sexually-transmitted infections, who smoke and frequently drink alcohol.

## Introduction

The Human Papillomavirus (HPV), a double-stranded DNA virus, is a risk factor for cervical cancer as types HPV 16 and 18 account for about 70% of all invasive cervical cancer worldwide (1). Cervical cancer is the fourth most common cancer among women, with estimated 604,000 new cases and 342,000 deaths globally (2). It is also the eighth most common cancer in women and men combined, contributing to 6·9% of new cancer cases diagnosed in 2020 (3, 4). The burden of cervical cancer in sub-Saharan African countries has become the highest globally, with over 90% leading to death due to limited access to early diagnosis and treatment (1–4).

In Botswana and Kenya, Tawe et al. showed that 83·7% of participants screened had high-risk (HR) HPV genotypes, while 16·3% had low-risk (LR) HPV genotypes (5). In a study involving 482 participants in Cameroun, Doh et al. reported a prevalence rate of 13·4% among the population (6). Kabuga et al. (2020) reported that Nigeria has an overall pooled HPV prevalence of 42% in the general population, with the highest (73%) and lowest (6%) prevalence in the Northwest and Southeast regions, respectively (7). HPV 16, 18, 31, 35, 52, and 58 were reported as the most predominant strains circulating among women in Nigeria with normal cervical cytology (7).

Numerous studies conducted outside of Nigeria reported that pregnant women have a higher prevalence of HPV infection than non-pregnant women (8–10). Few surveys in Nigeria assessed HPV prevalence across broad geopolitical areas while targeting key women subgroups. In Nigeria, Elukunki et al. showed a high prevalence of HPV infection among pregnant women aged 26-35 years (11). Farahmand et al. reported 42·6% infection among female sex workers (FSWs) in 33 countries (12). In Nigeria, Morhason-Bello et al. recorded 96% of HPV prevalence among FSW brothels in six different urban locations in Ibadan and identified HPV 35 as the most prevalent (13).

With exposure to the virus, 90% of the virus are cleared or eliminated within 12 to 24 months, especially in younger women. However, several factors influence this, e.g. HIV infection, as HIV-induced immunosuppression significantly impacts the HPV infection, persistence, and progression to cervical cancer (14). Various studies showed an increased prevalence and fatality of HPV infection among Women Living With HIV (WLWH), with Kabuga et al. reporting a pooled HPV-HIV coinfection of 37% (7, 15, 16).

Other predisposing factors for HPV infection include the early start of intercourse, multiple sexual partners, pregnancy, age at first pregnancy, and education (10, 17). Smoking, alcohol consumption, unprotected sex, occupation, and absence of circumcision reportedly contribute to HPV infections (18, 19).

There are reports on HPV prevalence in Nigeria, with ongoing interventions to increase HPV awareness among high-risk women groups, caregivers, sexually active individuals, secondary school students, and other cohorts (20–22). However, HPV prevalence among most at-risk women across Nigeria is a knowledge gap, and interventions may lack impact without guidance from knowledge of the stratified prevalence and associated sociobehavioural risk factors. The aim of the study is to determine the prevalence of HPV infection, especially among key women sub-groups across three geopolitical regions of Nigeria. It also sought to determine key socioeconomic and behavioural risk factors associated with HPV infection in those localities to generate evidence for action.

## Methods

### Study design

This study was a cross-sectional comparative study, obtaining ethical clearances from the Institutional Review Board of the Nigerian Institute of Medical Research (NIMR; IRB/20/008) and the Chinese Centre for Disease Control and Prevention (China CDC; No. 202111). Assuming the prevalence of HPV positive in women in Nigeria to be 18% with the significance level set at 5% and admissible error at 10%, sensitivity and specificity of self-sampling corresponding to medic-sampling were both 0·9, the formula below calculated the smallest sample size to be 192. All participants gave informed consent.


$$\underline{\text{N}}=\frac{\left[Z^{2}*S*(1-S)\right]}{P*E^{2}}$$

N = Minimum sample size; Z = normal deviate for two-tailed alternative hypothesis at a 95% level of significance = 1·96; S = Expected sensitivity and specificity of self-sampling method = 0·9; P= Prevalence of HPV among women in Nigeria = 18%; E - Margin of error (10%).

### Study population and recruitment criteria

We recruited study participants at the Nigerian Institute of Medical Research (NIMR) Antiretroviral therapy (ART) clinic and Outpatient clinic NIMR Lagos, NGOs outreach activities in Lagos and Owerri, Optimal Cancer Care Centre, Lagos and Delta State University Teaching Hospital. The NIMR ART clinic manages HIV-positive women, while its Outpatient clinic manages staff, their families, and patients attending our viral hepatitis and hypertension clinics.

Target women sub-groups in this study were female sex workers (FSWs), women seeking cancer-screening services (WCS), HIV-positive women, and pregnant women. The study period was approved in May 2020 and ended December 2021, cutting across the global COVID-19 lockdown and was impacted by the movement restrictions.

As such, the FSWs recruited here contacted their clients online, not brothel-based FSWs. After consenting, the participants had to abstain from sexual activity for 24 hours before sample collection. We excluded women who had undergone total hysterectomy, menstruating, and sexually inactive women. According to the manufacturer’s protocol, the haemoglobin from menstruating can affect PCR. We excluded women menstruating within the last three days before sample collection.

### Sample collection, preservation and questionnaire administration

Trained medics (doctors, nurses and healthcare workers) performed sampling at the healthcare facilities while self-sampling with pictorial guides was implemented at outreaches facilitated by two collaborating NGOs. The study utilised the Sansure Biotech Inc. and Beijing Genomics Institute (BGI) sample collection kits.

Self-administered semi-structured questionnaires obtained participants’ demographics, attitudes, practices, social habits, and risk factors, including age, educational levels, occupation, marital status, smoking and alcohol consumption, using hard-copy or online forms. The questionnaire included several redundant questions to infer participants’ status around uncomfortable questions people were prone to skip answering in public.

The testing laboratory in NIMR received the swab samples. The Sansure samples were stored at 2°C for immediate testing or at -20°C for more extended storage. Before analysis, the BGI sample cards were stored at -20°C or 80°C pending shipment to the BGI Service Laboratories (Hong Kong, China).

### Laboratory testing

The laboratory testing for the samples collected with Sansure kits was done at NIMR, while BGI service laboratories tested the samples collected with the BGI sample cards. Testing for HPV at NIMR utilised the Sansure 15 high-risk HPV Polymerase Chain Reaction-Fluorescence Probing kit (S3031E, Sansure Biotech Inc) on a Sansure Iponatic 96 system and later using a QuantStudio 5 (QS5) real-time PCR system.

The use of the 15 High-risk Human Papillomavirus Diagnostic Kit on an Iponatic 96 equipment required 20µl of the sample, 10µl lysis buffer, 30µl PCR-mix, and 2µl enzyme was added into predefined tubes as directed by the manufacturer’s protocol. Each sample was analysed on the Iponatic 96 system, performing sample preparation and real-time PCR testing within 28 minutes per sample.

According to the kit manufacturer’s instruction, the Sansure sample lysis reagent was used to release nucleic acids by adding an equal volume of lysis reagent and each sample to a reaction tube and incubating at room temperature for 10 minutes. Real-time PCR testing on the QS5 was performed using these raw lysates.

The Sansure 15 High-risk Human Papillomavirus Diagnostic Kit (S3031E) detects four targets, namely HPV 16, HPV 18, “Other High-Risk (OHR)”, and the human β-globin as an internal control. HPV18 was detected on the FAM channel, HPV16 on the CY5 channel, “Other High Risk” (OHR) HPV on the ROX channel, and the internal control on the VIC/HEX channel. If the internal control is undetected, the assay returns an “invalid” result. The internal control is a human marker, thus controlling for proper sample collection, extraction, and amplification.

Following a successful run, possible results include HPV negative or positive for HPV 16, HPV 18, “Other High Risk” (OHR), or any mixture of the three. The assay has a cyclic threshold (Ct) of ≤ 40 for the internal gene and ≤ 39 for HPV targets.

The BGI service laboratory utilised a PCR-NGS testing workflow to detect 14 high-risk HPV strains in samples collected using a special DNA-collection card to hold samples from the vaginal/cervical swab.

### Statistical analysis

Epi info 7® (US, CDC) was used for data analysis, applying chi-squared, ANOVA Fisher exact, and Kruskal-Wallis tests as appropriate, depending on the data distribution.

## Results

Nine hundred eighty-five samples were collected from unique study participants and tested. Thirty (30/985) had invalid results, while 955 (955/985) had valid results. Seventeen participants didn’t fill out the questionnaires, but most completed the questionnaire (968/985). However, many sections were left blank, primarily by those who filled out the hard-copy forms. The questionnaire helped obtain information about the participants regarding their socioeconomic status and behavioural patterns, summarising the critical responses in **Table 1**.

**Table 1:** Sociodemographic characteristics and key knowledge indicators of the study participants.

S/N	Category	Data	Frequency (Number [Percent])	HPV Positive (Number [^#^Percent])
**1**	Age	< 30 years	186 (18·9%)	74 (39·8)
		≥ 30 years	717 (72·8%)	141 (19·7)
		Don't Know/No Response	82 (8·3%)	15 (18·3)
**2**	Marital Status	Single	230 (23·4%)	76 (33·8)
		Married/Cohabit	601 (61·0%)	113 (19·6)
		Widowed/Divorced/Separated	99 (10·0%)	30 (30·3)
		Don't Know/No Response	55 (5·6%)	19 (35·9)
**3**	Educational Status	None	10 (1·0%)	6 (60·0)
		Primary	54 (5·5%)	20 (37·0)
		Secondary	273 (27·7%)	83 (30·4)
		Tertiary	584 (59·3%)	111 (19·0)
		Other (unknown, unorthodox)	64 (6·5%)	18 (28·1)
**4**	Ever tested for HIV?	No	101 (10·3%)	22 (21·8)
		Yes	756 (76·7%)	183 (24·2)
		Don't Know/No Response	128 (13·0%)	33 (25·8)
**5**	HIV Status	Negative	13 (1·3%)	10 (76·9)
		Positive	205 (20·8%)	58 (28·3)
		Don't Know/No Response	767 (77·9%)	170 (22·2)
**6**	Ever contracted *STIs?	No	484 (49·1%)	108 (22·3)
		Yes	341 (34·6%)	92 (27·0)
		Don't Know/No Response	160 (16·2%)	38 (23·8)
**7**	Heard about HPV	No	479 (48·6%)	127 (26·5)
		Yes	365 (37·1%)	77 (21·1)
		Don't Know/No Response	141 (14·3%)	34 (24·1)
**8**	HPV Preventable?	No	44 (4·5%)	9 (20·5)
		Yes	334 (33·9%)	70 (21·0)
		Don't Know/No Response	607 (61·6%)	159 (26·2)
**9**	HPV treatable?	No	43 (4·4%)	9 (20·9)
		Yes	338 (34·3%)	67 (19·8)
		Don't Know/No Response	604 (61·3%)	162 (26·8)
**10**	Heard about HPV Vaccines?	No	286 (29·0%)	64 (22·4)
		Yes	266 (27·0%)	56 (21·1)
		Don't Know/No Response	433 (44·0%)	118 (27·3)
**11**	Take HPV Vaccine?	No	734 (74·5%)	178 (24·3)
		Yes	61 (6·2%)	15 (24·6)
		'Don't Know/No Response	190 (19·3%)	45 (23·7)

*STIs, Sexually Transmitted Infections. ^#^Percentage of the frequency per line option.

About 37% of the women confirmed they had heard about HPV before, resulting in poor knowledge of prevention and treatment for HPV infections (**Table 1**). More importantly, even fewer (27%) knew there were vaccines against HPV, and very few (6%) confirmed vaccination with HPV vaccines. Among the women (n=60) vaccinated against HPV, most were from the cohort seeking cancer-screening services (n=42), the general population (n=17) and FSW (n=1).

The three study sites were in Delta state (South-South), Imo state (Southeast) and Lagos state (South-West). Out of 955 participants with valid tests, 238 (24·9%) were HPV positive, while 717 were HPV negative. Each site had varied HPV prevalence (**Table 2**). A few participants skipped the site identifier response in the questionnaire, and we could not ascertain their study sites precisely. Four hundred fourteen samples were tested using the BGI workflow, 503 were tested using the Sansure workflow, and 68 were tested using both methods.

**Table 2:** HPV prevalence across the three collection sites/states.

Site/States	HPV positive (n)	HPV negative (n)	Invalid tests (n)	Total (n[%])	HPV prevalence (%)
Delta-SS	46	84	0	130 (13·2)	35·4
Imo-SE	37	214	26	277 (28·1)	13·4
Lagos-SW	145	409	4	558 (56·7)	26·0
*No Response	10	10	0	20 (2·0)	50·0
**Total**	**238**	**717**	**30**	**985**	**^#^24·9**

*Did not include a response in the questionnaire. **^#^** Invalids were excluded (238/955).

**Table 3** shows the HPV prevalence among the target women subgroups, including HIV-positive women, online FSWs, Pregnant women, and women seeking cancer-screening services (WCS). Among those without clear site identification, 50% were HPV positive. Within the target women sub-groups, HPV prevalence was the highest among pregnant women, followed by online FSWs, women seeking cancer-screening services, and HIV-positive women (**Table 3**). Most participants were among the general women population (401/955) recruited during public community women’s health outreaches and had an HPV prevalence of 17·2 (69/401).

**Table 3:** HPV status among the women population.

Population Sub-group	HPV Positive (n)	HPV Negative (n)	Invalid test (n)	Total (n[%])	HPV Prevalence (%)
General population	69	332	28	429 (43·6)	16·1
^#^HIV-Positive women	39	129	2	170 (17·3)	22·9
^&^WSC	39	97	0	136 (13·8)	28·7
Pregnant women	44	78	0	122 (12·4)	36·1
FSWs	37	73	0	110 (11·2)	33·6
*No Response	10	8	0	18 (1·8)	55·6
Total	238	717	30	985	24·9

*No response in questionnaire response. ^&^WSC, Women Seeking Cancer-screening services. **^#^** Few women within the other sub-groups were also HIV-positive from their questionnaire response.

The socioeconomic and behavioural risk factors from their questionnaire responses were evaluated for association with HPV infection. First, HPV status was compared to the women’s pregnancy status. Out of 862 women who responded, 834 had valid results, while 28 women had invalid results. Twenty-three percent (172/724) of women who were ever pregnant were HPV positive, while 32% (35/110) of those not pregnant were HPV positive (p=0·04). The odds ratio of the pregnant women being HPV positive was 0·67 (95% CI: 0·43-1·03).

Among the participants, there were Christians (87%), Muslims (8%) and unknown religions (5%), but their religion was not associated with their HPV status (p=0·44). HPV infection varied across educational levels, with the highest prevalence among women without education (6/8 [75%]) and the least among women with tertiary education (111/563[19·7%]; p=0·00).

HPV prevalence was also significantly different across the occupational groups, with infection highest among those working as unskilled personnel (p=0·003). HPV infection cross-stratified by educational levels and occupation showed HPV infection was highest among those without education (3/3 [100%]) and those with primary education (12/22 [54%]) working as unskilled personnel.

The prevalence of HPV, according to the women’s marital status, showed the infection was highest among women with unknown marital status (35·9%), followed by single women (33·8%), widowed/divorced/separated women (30·3%), and married/cohabiting women (19·6%) (p=0·00).

HPV infection status cross-tabulated with age showed the HPV-positive women were younger (Median= 34 years; Interquartile range [IQR]: 27-40 years) than the HPV-negative women (Median= 38 years; IQR: 32-44 years) (p< 0·000). Cross-stratifying HPV infection and marital status by age (under 30 years and ≥ 30 years) gave a significant difference only for women ≥ 30 years, with infection highest among widowed/divorced/separated women (32·6%) (p=0·006).

HPV infection was significantly higher among women who take alcohol (**Table 4**; p<0·000), with odds of 1·7 (95% CI: 1·2-2·4), especially those who took alcohol last week (33·8%) compared to those who took it six months ago (15·6%; p=0·026). Similarly, infection was higher in women who take strong spirits (37·5%) compared to women who take low-alcohol drinks (26·8%; p<0·000). In addition, infection was higher in women who take 4-13 bottles/week (42·9%) and least in those who take alcohol occasionally (19·2%; p=0·027).

**Table 4:** Behavioural risk factors associated with HPV infection.

Ever take alcohol?	HPV positive (n)	HPV negative (n)	Total (n)	HPV prevalence (%)	Odds for HPV infection
** Yes**	158	404	562	28·1	1·7 95% CI: 1·3 - 2·4 P < 0·000
** No**	66	291	357	18·5	
** Total**	224	695	919	24·4	
Smoke?	HPV positive (n)	HPV negative (n)	Total (n)	HPV prevalence (%)	Odds for HPV infection
** Yes**	21	27	48	43·8	2·6 95% CI: 1·4 – 4·6 P = 0·001
** No**	203	667	870	23·3	
** Total**	224	694	918	24·4	
Take alcohol + smoke	HPV positive (n)	HPV negative (n)	Total (n)	HPV prevalence (%)	Odds for HPV infection
** Yes**	18	24	42	42·9	2·4 95% CI: 1·3 – 4·4 P = 0·005
** No**	220	693	913	24·1	
** Total**	238	717	955		

In addition, HPV infection was significantly higher among women who smoke (**Table 4**), with an odds ratio of 2·6 (95% CI: 1·4 - 4·6). More importantly, women who both take alcohol and smoke have a higher HPV infection rate compared to others who do not both smoke and take alcohol.

**Figure 1:**
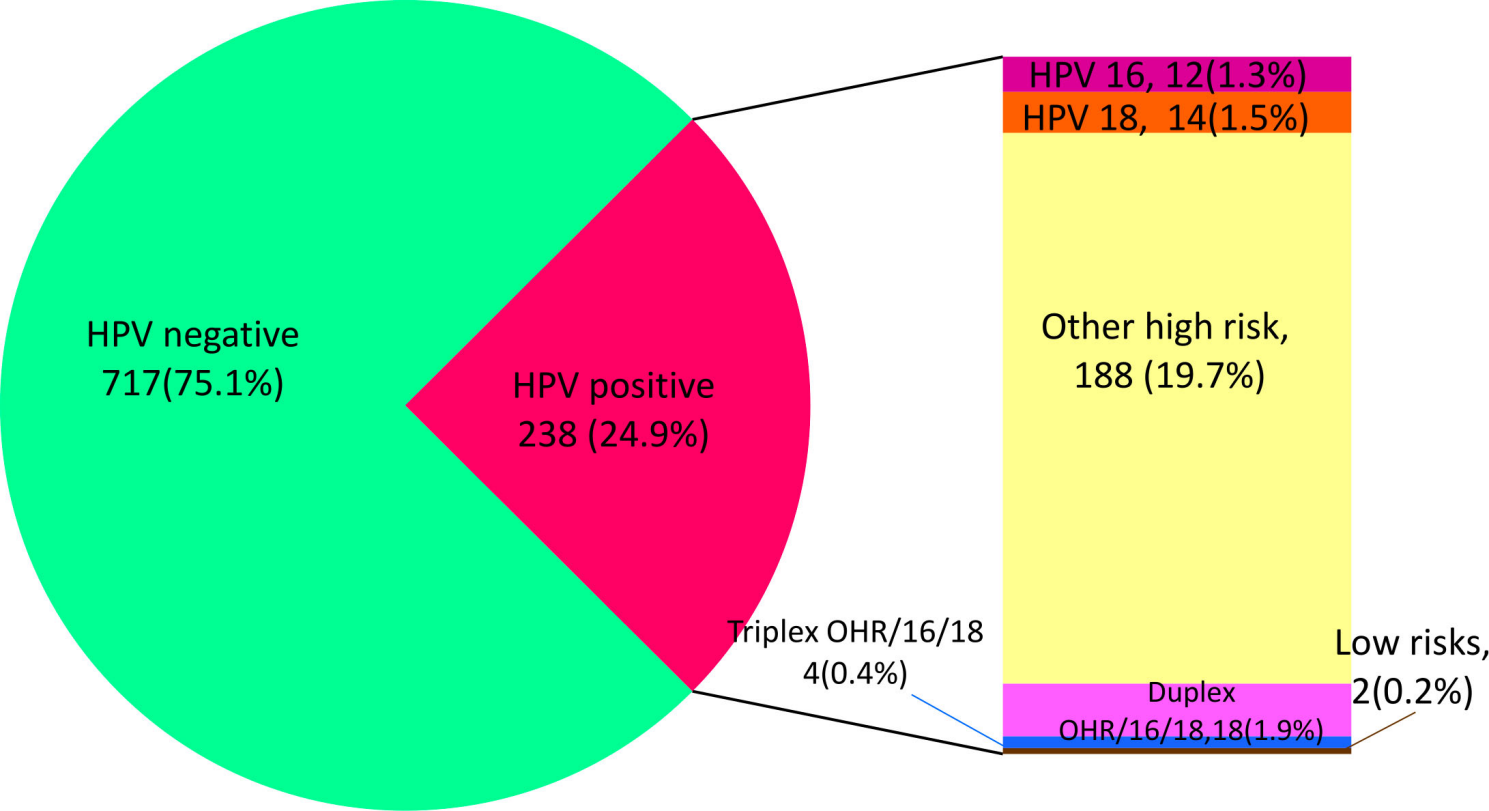
Prevalence of HPV and strains detected.

HPV strains detected among those infected were HPV 16, 18, Low Risk (LR) and Other High-Risk groups (OHR) (**Figure 1**). The OHR HPV strains had the highest prevalence (78·9%), followed by HPV18 (5·9%) and HPV16 (5·0%). Eighteen cases of duplex infection with OHR and 16 or 18 were detected, along with four cases of triple infection with OHR, HPV16 and HPV18.

## Discussion

This cross-sectional study determined the prevalence of HPV among key women sub-groups and the socioeconomic and behavioural factors associated with HPV infection among women across three geopolitical zones of Nigeria. The study confirmed sexually active women and women who smoke and take alcohol are at higher risk of infection. Poor awareness of HPV prevention and treatment exists.

Infection levels are similar among those vaccinated and those not vaccinated, questioning the efficacy of the vaccination and its implementation process. We found little awareness about HPV, calling for more sensitisation, especially in rural areas with limited access to information and media. Some facilities provide vaccination services and can reach rural areas to increase vaccination coverage. More importantly, can the available vaccines guarantee protection from the dominant strains circulating locally? This study detected HPV 18, 16, low-risk (LR) and several other high-risk (OHR) strains. The high prevalence of OHR strains calls for further research into their possible association with cervical cancer in Nigeria since most vaccines currently available in Nigeria guarantee protection from HPV 16 and 18. Future research can investigate this and the association of OHR strains in cervical cancers, especially in cases of either duplex or triplex infection involving the OHR strains and HPV16 or HPV18.

Pregnant women were the target subgroup with the most infection, though their questionnaire response did not collaborate with this finding. Earlier studies had reported higher HPV prevalence among sexually active and pregnant women (8–10, 23). One common factor among the women subgroups with high infection is being sexually active. Notably, all the subgroups are potential groups at higher risks of infection and had infection rates above that of the general women population. This finding suggests and confirms being sexually active, especially with multiple partners, is a more significant risk factor than possible immunodeficiency from HIV infection. Pradhan et al. suggested hormonal changes and immune response during pregnancy might influence the persistence of HPV infection, while Siristatidis et al. (2018) attributed low HPV prevalence in men to the absence of such hormones (10, 24). The most educated women being the least infected indicates that knowledge and awareness campaigns can help if implemented and possibly target pregnant women and women with little education in unskilled jobs. The disparity in HPV infection according to educational and employment status emphasises the power of knowledge and empowerment. It aligns with Believe et al. claim that education is a crucial factor that enlightens the danger of diseases (25). Our report shows HPV prevalence is inversely proportional to the education level of the women, suggesting interventions to increase awareness about cervical cancer and prevention strategies might be very effective. This study also reported high infection in moderately educated women in unskilled jobs, with two possible implications. It may indicate the knowledge is not engendering protective action. On the other hand, as unskilled people often earn little, the desperation for survival can encourage indulgence in risky sexual behaviours and predispose them to infections. Interventions to produce more knowledgeable and empowered women, especially financial empowerment, may help women better protect themselves and reduce infection.

Married or cohabiting women exhibited a lower HPV infection, assuming having a steady sexual partner is responsible, then those with multiple sexual partners are at higher risks for HPV infection (26). Single and divorced/separated/widowed women may be open to several sexual partners, while FSWs act as reservoirs, transmitting the infection to their clients. Similarly, being open to multiple sexual partners could have resulted in younger women exhibiting higher infection rates. However, this finding seems contrary to the norm of more infection in older women. Stratifying infection and marital status by age gave the expected result that sexually active and older women had more infections. These assumptions need empirical verification to plan and implement appropriate interventions.

A third of the women had contracted STIs earlier, while a quarter was HIV-positive. And infection among the HIV-positive cohort was higher than in the general population. HIV is known to reduce the immune cells, paving the way for other infections (27, 28). This interference with the immune system could be responsible for the high HPV prevalence reported among HIV-positive women. Targeting HIV-positive women and those presenting with STIs with HPV awareness, testing and cancer screening may be worthwhile.

The study replicated the association of smoking and alcohol intake with HPV infection. More importantly, we show the two behavioural lifestyles appear synergistic in their association with HPV infection. Mbulawa et al. documented how alcohol increases exposure to HPV (29). HPV infection correlated positively with the use of strong alcohol, current alcohol use and the volume consumed in this study. Higher infection in women who consume strong spirits though less frequently, suggests the nature of the alcoholic drink, not the quantity consumed, should be considered, suggesting risky sexual behaviour under the influence of alcohol could be the culprit. In this study, we report the synergistic influence of taking alcohol and smoking on HPV infection. We found that women who drank and smoked had more infections, with twice the odds of being infected with HPV. Thus, it further indicates risky sexual behaviour while intoxicated as the possible infection causes. We postulate engaging in these lifestyles may increase risky sexual practices, leading to higher HPV infection rates. These women could be targets for risk reduction programmes, especially current and heavy consumers of strong alcoholic drinks.

Another exciting finding is the high prevalence of HPV infections among women seeking cancer-screening services using traditional technologies. The high prevalence of HPV infection among this cohort may indicate the need for general screening with HPV DNA testing to identify infected women early, hopefully before the occurrence of cellular changes the routine methods detect and improve the prognosis.

Prevalence for the three geopolitical zones may be biased for South-west (Lagos state) and South-south (Delta state). Delta State recruited predominantly pregnant women, while Lagos State had the most HIV-positive women. Thus, the higher HPV prevalence for these two regions may be biased by these sub-groups with a higher prevalence of HPV infection, as Anoruo et al. reported a higher prevalence in the Southeast region (30). These findings call for more surveillance though this study’s overall prevalence (25%) correlates with earlier reports. Finally, the high HPV prevalence among the general women population calls for more surveillance, increased awareness and vaccination programmes possibly targeting key cohorts. The low vaccination uptake of 6% calls for urgent intervention to boost access to vaccines and coverage among eligible women.

### Limitations

The strengths of this study include the spread across geopolitical zones in Nigeria, the use of validated self-collection kits and, ultimately, an electronic consent form and questionnaire. The electronic questionnaire allowed the women to answer sensitive questions comfortably in their homes. Within 5-10 minutes of arriving at the facility, they could collect their sample, submit this, and be on their way. Depending on the site, validated self-sampling and medic-sampling protocols were implemented, so we do not expect an impact on the results and findings. However, the study first used hard-copy questionnaires, and many women skipped several questions and identifiers in their questionnaires while filling this out publicly. A high infection rate was recorded among women without explicit site identifiers meaning many were suspicious or may know their status and avoided the study identifiers and some questions. So they are all included in the analysis. However, the numbers were small and would not significantly affect the findings. Secondly, two different sample collection kits (Sansure and BGI) were used to collect samples and perform the HPV DNA test. As both tests are approved by the Chinese regulator, we believe there will be negligible variation arising from use of two different workflows. Finally, the study did not cover all six geopolitical zones in Nigeria and may be biased in estimating prevalence for some zones.

## Data availability statement

Deidentified study data underpinning this manuscript can be made available upon request from the corresponding author after publication and subject to the approval of the institutional ethics board and a signed data access agreement.

## Ethics statement

This study was conducted according to the guidelines in the Declaration of Helsinki, and all participants gave their consent. The Institutional Review Board of the Nigerian Institute of Medical Research approved the study (IRB/20/008) and the Chinese Centre for Disease Control and Prevention (China CDC) (No. 202111).

## Author contributions

CKO: Conceptualisation, Data curation, Formal Analysis, Funding acquisition, Investigation, Methodology, Project administration, Resources, Supervision, Validation, Visualisation, Writing – original draft, Writing – review & editing. NF: Conceptualisation, Data curation, Formal Analysis, Funding acquisition, Investigation, Methodology, Project administration, Resources, Visualisation, Writing – original draft, Writing – review & editing. AM: Data curation, Formal Analysis, Investigation, Methodology, Validation, Writing – original draft, Writing – review & editing. MU: Investigation, Methodology, Writing – original draft, Writing – review & editing. RA: Data curation, Formal Analysis, Investigation, Methodology, Supervision, Validation, Writing – original draft, Writing – review & editing. II: Methodology, Validation, Writing – original draft, Writing – review & editing. GV: Formal Analysis, Investigation, Methodology, Supervision, Writing – original draft, Writing – review & editing. CO: Data curation, Methodology, Writing – original draft, Writing – review & editing. NO: Investigation, Methodology, Writing – review & editing. JS: Investigation, Methodology, Project administration, Supervision, Validation, Writing – review & editing. BA: Data curation, Formal Analysis, Investigation, Methodology, Project administration, Supervision, Writing – original draft, Writing – review & editing. TO: Data curation, Formal Analysis, Methodology, Writing – original draft, Writing – review & editing. LO: Investigation, Methodology, Supervision, Writing – review & editing. MA: Formal Analysis, Investigation, Methodology, Writing – original draft, Writing – review & editing. PE: Investigation, Methodology, Writing – review & editing. OS: Data curation, Investigation, Methodology, Writing – review & editing. JO: Investigation, Methodology, Validation, Writing – review & editing. OL-P: Data curation, Investigation, Methodology, Writing – review & editing. JOS: Conceptualisation, Supervision, Writing – review & editing. GO: Investigation, Methodology, Supervision, Writing – review & editing. EN: Data curation, Investigation, Methodology, Writing – review & editing. AD: Investigation, Methodology, Writing – review & editing. OO: Investigation, Methodology, Supervision, Writing – review & editing. IO: Methodology, Supervision, Validation, Writing – review & editing. X-PD: Conceptualisation, Funding acquisition, Project administration, Resources, Validation, Writing – review & editing. OE: Data curation, Formal Analysis, Investigation, Methodology, Resources, Supervision, Validation, Writing – review & editing.
